# Self‐Immolation in South Asia: Epidemiology, Motives, and Sociocultural Contexts

**DOI:** 10.1002/puh2.70268

**Published:** 2026-05-05

**Authors:** S. M. Yasir Arafat, Sadeed Hossain, Sujita Kumar Kar

**Affiliations:** ^1^ Department of Psychiatry Bangladesh Specialized Hospital Dhaka Bangladesh; ^2^ Department of Public and Community Health Faculty of Medicine and Health Sciences Frontier University Garowe Garowe Somalia; ^3^ Biomedical Research Foundation Dhaka Bangladesh; ^4^ Department of Psychiatry King George's Medical University Lucknow Uttar Pradesh India

**Keywords:** epidemiology, self‐immolation, social context, South Asia, suicidal burning

## Abstract

Self‐immolation is a neglected public health and social problem in South Asia. This review focuses on epidemiological patterns, sociodemographic characteristics, motives, and psychosocial correlates associated with self‐immolation in South Asian countries. A narrative review was conducted using PubMed, Scopus, Google Scholar, BanglaJOL, and Google. A search for original peer‐reviewed studies, published in English between 1990 and 2024, on self‐immolation in eight South Asian countries, yielded a total of 11 studies. Six studies were from India; no study from Bangladesh, Bhutan, Maldives, and Nepal was found. The studies’ sample sizes varied considerably (single case report to 260 participants). Self‐immolation was mostly likely among females between age 16 and 30 years. Oppression, abuse, and physical violence by family members were the most commonly motives of female self‐immolators. Kerosene and open flames were the most frequently used means. Fatality rates ranged from 70% to 100%. Studies are warranted to explore the psychological, cultural, and human rights‐violations factors contributing to female self‐immolation and to identify female‐centered and culture‐ and human‐rights‐grounded preventive strategies.

## 1 Introduction

Suicide is one of the most enduring psychosocial problems worldwide. People die by suicide in various ways including hanging, poisoning, firearms, and some other methods with regional variations like self‐immolation [1]. The World Health Organization reported that every year more than 720,000 people lose their lives due to suicide, where nearly three‐fourths (73%) of all suicide deaths occur in low‐ and middle‐income countries (LMICs) [2]. There is a disparity in the incidence of self‐immolation between high‐ and low‐income countries. Approximately, only 1% of suicides in high‐income countries (HICs) (Western Europe and the United States) occur by self‐immolation, whereas around 40% of suicides in low‐income countries occur by self‐immolation [3, 4]. This represents HICs account for only a minority of self‐immolation cases compared to the LMICs where there is a dearth studies on the phenomenon. Various factors like individual's psychological distress and several external factors; broader societal oppression such as economic and social discrimination towards women, coercive marital structures, exposure to gender‐based violence, restrictions on women's autonomy and mobility, limited access to education and employment, and inadequate access to mental health care, as well as economic crisis and political protest, are associated with self‐immolation [3, 4, 5, 6].

Approximately 2 billion people live in eight countries (Afghanistan, Bangladesh, Bhutan, India, Maldives, Nepal, Pakistan, and Sri Lanka) in South Asia with LMIC backgrounds, making up around one‐fourth of the world's population [7, 8, 9]. In South Asian countries such as India, Pakistan, Afghanistan, and Sri Lanka, self‐immolation had been linked to violence against women like forced marriage, dowry‐driven violence, protest suicides, and social oppression for women [10, 11, 12]. These variations highlighted how people might attempt self‐immolation due to an act of personal distress as well as express a form of socio‐political environment.

Prior systematic reviews of suicide by self‐immolation focused on the sociocultural context of protest suicide by self‐immolation among Muslim women, of self‐immolation in women and men globally, and of protest self‐immolation among women in South and Central Asia [12, 13, 14]. Another study focused on case studies in South Asia [10]. This review revisits evidence on self‐immolation across South Asian countries, emphasizing its epidemiology, motivations, and sociocultural contexts. It confirms prior reviews’ [10, 12, 13, 14] findings regarding common motives, patterns, and psychosocial links related to self‐immolation, along with key insights for prevention and policymaking.

Consequently, this review consolidated existing evidence on self‐immolation in South Asian countries, emphasizing its epidemiology, motivations, and sociocultural contexts. By analyzing multiple studies, the review uncovered common motives, patterns, and psychosocial links related to self‐immolation, along with key insights for prevention and policymaking. Recognizing these complex risk factors is crucial for creating culturally and gender‐sensitive strategies to reduce this self‐immolation in the region.

## 2 Methods

### 2.1 Study Design

This narrative review was conducted to summarize existing evidence on self‐immolation in South Asian countries.

### 2.2 Search Strategy

A comprehensive literature search was performed in electronic databases including PubMed, Scopus, Google Scholar, BanglaJOL, and Google. The search covered all articles published in English up to October 12, 2025. Keywords and combinations of Medical Subject Headings (MeSH) used were (“self‐immolation” OR “self immolation” OR “self‐immolate” OR “self immolate” OR “self‐burning” OR “self burning” OR “self‐inflicted burn” OR “self inflicted burn” OR “self burn” OR “self immolation”) AND (“Afghanistan” OR “Bangladesh” OR “Bhutan” OR “India” OR “Maldives” OR “Nepal” OR “Pakistan” OR “Sri Lanka” OR “South Asia” OR “South Asian”).

Free text words were also used to search in Google Scholar, BanglaJOL, and Google. A snowball approach was used by screening the reference lists of selected articles to identify additional relevant studies with special attention to previously published evidence [12, 13, 14].

### 2.3 Inclusion Criteria

This review included all peer‐reviewed publications that were original research articles, case series, case reports, and letter to the editors reporting primary data. Both fatal and nonfatal self‐immolation cases were considered, with no restrictions on publication year, to ensure a proper understanding of trends and contributing factors over time.

### 2.4 Exclusion Criteria

Studies were excluded if they primarily reported accidental burns or non‐suicidal injuries, as these do not represent self‐immolation. Studies conducted outside South Asia were excluded to maintain the regional focus of the review. Additionally, editorials, book chapters, review articles, and letters to the editors without reporting primary data were excluded to avoid duplication of data.

### 2.5 Data Extraction and Synthesis

For each included study, relevant data were systematically extracted and tabulated in Microsoft Excel, 2016. This study aimed to focused on epidemiological patterns, sociodemographic profiles, motives, psychological factors, and sociocultural contexts of self‐immolation in eight South Asian countries. The extracted information included the country and year of the study, study design, sample size, characteristics of the study population (age and sex), motives, methods or means used, and the outcomes (fatal or nonfatal). Additional details were collected on key findings, prevention strategies, and/or recommendations proposed. Findings were summarized descriptively. No statistical or meta‐analytic methods were used because of the heterogeneity and small number of available studies.

### 2.6 Ethical Considerations

As this review utilized publicly available data and did not involve human participants directly, ethical approval was not required.

## 3 Results

### 3.1 Study Characteristics

The search identified 16 articles. A total of 11 studies published between 1990 and 2024 were included after excluding two book chapters, two review articles, and one qualitative study [10, 11, 15, 16, 17, 18, 19, 20, 21, 22, 23]. The majority of articles were published from India (*n* = 6) [10, 15, 16, 17, 18, 19] followed by Pakistan (*n* = 2) [20, 21], Sri Lanka (*n* = 2) [22, 23], and Afghanistan (*n* = 1) [11]. No study was noted from Bangladesh, Bhutan, Maldives, and Nepal. Among the 11 studies, 5 articles were noted as hospital‐based descriptive or observational designs [11, 13, 15, 18, 21], 2 articles were comparative [19, 23], and one was cross‐sectional study [17]. There was one case report [20], one case series [16], and one letter from editors [22]. Sample size ranged from a single case report [20] to 260 participants [17].

### 3.2 Demographic and Female–Male Patterns

Self‐immolation was mostly attempted by females, representing 65%–100% of participants in most reports. Mean age ranged from late adolescence to early adulthood, with most studies reporting peak prevalence between 16 and 30 years. The mean or median age in included studies varied between 18.2 [18] and 31 years [17], underscoring the predominance of young adult self‐immolators. Notably, Wagle et al. [19] in India reported that all the self‐immolators were under 35 years [16].

### 3.3 Key Domain Studied

Most studies (*n* = 8) explored sociodemographic and phenomenological aspects of self‐immolation [11, 12, 14, 16, 18, 19, 20, 21], only two studies examined psychological correlates [18, 20], and one study reported the treatment process and outcomes of a patient after self‐immolation [15]. Collectively, these studies highlighted consistent regional patterns of female predominance and early adulthood onset associated with self‐immolation across South Asia (Table 1).

**TABLE 1:** Characteristics of studies on self‐immolation in South Asia.

S. no.	Study	Country	Study design	Sample size	Study sample	Sex distribution	Age distribution	Key domain studied
1	Raj et al. [11]	Afghanistan	Descriptive	77	Afghan women and girls with burn injuries	Female (100%)	12 years and older (majority 16–19 years; 55%)	Sociodemography and phenomenology
2	Natarajan [17]	India	Cross‐sectional	260	Burn patients from a government hospital	Female—65% Male—35%	Mean (±SD): 31 ± 16 years	Sociodemography
3	Mahla et al. [16]	India	Case series	4	Burn patients from a hospital	Female—25% Male—75%	Mean ± SD: 21 ± 2.45 years	Sociodemography and phenomenology
4	Ganesamoni et al. [15]	India	Observational	85	Burn patients from a hospital during	Female—64.7% Male—50.9% Not reported—2.4%	Mean ± SD: 24.58 ± 1.01 years	Sociodemography and treatment
5	Kumar [10]	India	Descriptive	32	Married women	Female—100%	Age Range: 16 → 40 years	Sociodemography and phenomenology
6	Singh et al. [18]	India	Descriptive	22	Burn patients	Female—45.5% Male—54.5%	Mean 18.2 years (range 13–36 years)	Sociodemographic and psychology
7	Wagle et al. [19]	India	Comparative	20	Burn patients	Females > Males	<35	Sociodemography
8	Saaiq and Ashraf [21]	Pakistan	Descriptive	93	>14 years who presented as cases of burn suicides and attempted suicides	Female—80.6% Male—19.4%	Mean ± SD: 26.89 ± 6.1 years (range 15–52 years)	Sociodemography
9	Amjad and Zahra [20]	Pakistan	Case report	1	Patient with major burns	Female—100%	35 years	Sociodemographic and psychology
10	Laloë and Ganesan [23]	Sri Lanka	Comparative	87	Burn patients	Female—79.3% Male—20.7%	Median: 27 years	Sociodemography
11	Fernando et al. [22]	Sri Lanka	Letters to editor	51	Suicidal deaths from burns	Female—76.5% Male—23.5%	Not reported	Sociodemography

### 3.4 Social Context of Self‐Immolation

As shown in Table 2, women's exposure to spousal abuse, coercive marital control, and domestic violence by family members emerged as the most commonly reported social context for self‐immolation. Although several studies used terms such as “marital conflict” or “domestic disputes,” closer examination of the reported circumstances reveals patterns of gender‐based violence, forced marriage, dowry‐related abuse, and sustained oppression within the household, as noted in previous studies [12, 13, 14].

**TABLE 2:** Factors, methods, outcomes, and psychosocial correlates reported in studies on self‐immolation in South Asia.

S. no.	Study	Social context	Means	Outcome	Psychological context	Prevention/Recommendations
1	Raj et al. [11]	Forced/Child marriage (29%), “badal” exchange marriage (18%), abuse from in‐laws (16%), spousal violence, family conflict, and poverty	Kerosene	80% fatal	Not reported	Strengthen enforcement of women's rights laws, programs preventing violence against women, and improve social support and women's education
2	Natarajan [17]	Domestic violence, marital discord, poverty, unemployment, infidelity, and mental health	Open flame 23%, Kerosene 70%, and stove 0.4%	Fatal—80%	Psychological distress	Gender‐sensitive prevention, social service, and legal system involvement
3	Mahla et al. [16]	Unemployment, job dissatisfaction, unmet student demands, desire for fame, alcohol/tobacco use, and poor social support	Kerosene		Personality disorders (borderline, narcissistic, histrionic, and schizoid)	Not reported
4	Ganesamoni et al. [15]	Not reported	Not reported	Fatal—83.5%	Not reported	Not reported
5	Kumar [10]	Dowry, joint families, social pressure, and low education	Match stick 40.6%, wood cooking 9.4%, Kerosene stove 34.4%, Kerosene lamp 9.4%, and gas cooking 6.3%	Fatal—100%	Not reported	Increase women's education, anti‐dowry legislation, and awareness campaigns
6	Singh et al. [18]	Protest against government reservation policy	Not reported	Fatal—27.3%	Psychopathology, dysthymia, and family history of suicide/alcohol dependence	Not reported
7	Wagle et al. [19]	Unemployment, marital conflict, in‐law conflict, dowry issues, financial problems, lack of child/son, illness, death in family, alcohol abuse in family, and joint family stress	Not reported	Fatal—10%	Stressful life events	Not reported
8	Saaiq and Ashraf [21]	Marital conflicts (61.29%), failed love affairs (9.67%), political protests (2.15%), depression/alcohol (2.14%), illiteracy, and rural background	Not reported	Fatal—84.9%	Not reported	Not reported
9	Amjad and Zahra [20]	Not reported	Locked bedroom and set self on fire	Not reported	Postpartum psychosis	Screen pregnant women for perinatal psychiatric disorders
10	Laloë and Ganesan [23]	Marital discord, domestic dispute, alcohol, and low education	Kerosene	Fatal—70%	Psychiatric illness	Not reported
11	Fernando et al. [22]	Marital discord, family conflicts, broken relationships, low education, and socioeconomic stress	Not reported	Fatal—100%	Psychosis	Not reported

In Afghanistan, Raj et al. [11] found forced or child marriage (29%), exchange/*badal* marriage (18%), and abuse from in‐laws (16%) as primary risk factors of self‐immolation. Similarly, in Pakistan, Saaiq and Ashraf [21] reported marital conflicts (61.3%) and failed love affairs (9.7%) were the most commonly reported circumstances preceding self‐immolation. Indian studies frequently cited dowry‐related issues, marital discord, and financial distress [16, 17, 18, 19], whereas psychological disorders and protest motivations were less common but present [18].

Family‐related pressures within joint family systems and economic dependency were also recurrent risk factors [16, 21], and these particularly show the structural vulnerability of women arising from coercive family systems, economic dependency, and lack of legal and social protection from violence. In Afghanistan and India, self‐immolation was often framed as a symbolic protest against oppression [11, 16]; however, in Sri Lanka and Pakistan, it was intertwined with marital discord and psychosocial distress [20, 21, 22, 23].

This variation may reflect differences in sociopolitical history, gender norms, and the symbolic meanings attached to self‐harm across these societies. In Afghanistan and parts of India, self‐immolation has historically functioned as a highly visible form of protest, particularly for women facing systemic oppression or rigid patriarchal control [10, 11]. In these contexts, self‐immolation may represent not only personal suffering but also a symbolic act of protest, used to express collective frustration and to draw public and political attention to broader social and structural injustices [12, 13, 14].

In contrast, in Sri Lanka and Pakistan, self‐immolation is more often linked to private life circumstances, particularly family relationships, marital conflict, and psychological distress. Strong social expectations around marriage and gender roles, together with few socially acceptable ways to express emotional suffering, may lead individuals to internalize distress rather than express it through public or political protest [20, 21, 22, 23].

These findings suggest that self‐immolation in South Asia frequently occurs within a broader context of structural gender inequality, family conflict, and socioeconomic stress. For many women, the act appears to occur in environments characterized by limited autonomy, restricted social support, and persistent domestic oppression [12, 13, 14].

### 3.5 Psychological Context of Self‐Immolation

Psychological factors were reported in several studies, although they were less frequently documented than social determinants. A small number of studies described underlying psychiatric conditions among individuals who attempted self‐immolation. Reported conditions included personality disorders, psychological distress, dysthymia, psychosis, substance dependence, and postpartum psychosis [13, 14, 15, 17, 19, 20].

For example, Singh et al. [18] identified various psychiatric manifestations among individuals who attempted self‐immolation, including substance dependence and mood disorders. A case report from Pakistan described self‐immolation associated with postpartum psychosis [20]. These findings suggest that while structural social conditions often create the environment in which self‐immolation occurs, individual psychological vulnerabilities may further increase the risk.

Overall, the available evidence indicates that self‐immolation in South Asia reflects a complex interaction between social adversity and psychological distress. However, the relatively small number of studies assessing mental health factors suggests that psychiatric aspects of self‐immolation remain under‐researched in the region.

### 3.6 Methods and Outcomes

Kerosene and open flames were the most frequently used means of self‐immolation across nearly all studies, either via stoves, lamps, or direct ignition [11, 20, 21]. These similar findings underscore that the easy household accessibility of inflammable materials might have made self‐immolation easier.

Fatality rates were high, ranging from 70% to 100% in most studies. The highest mortality (100%) was observed among dowry‐related deaths [10] and all recorded deaths in Sri Lankan medicolegal reviews [22]. Similarly, treatment‐focused study also revealed high mortality [15]. However, studies that were conducted on focusing on protest and psychological cases reported lower mortality [16, 18, 19, 20]. Overall, self‐immolation resulted in death in approximately 70%–85% of reported cases across South Asia (Table 2).

### 3.7 Cross‐Country Patterns and Variations in Self‐Immolation

Analysis of the included studies revealed some cross‐country differences and similarities in self‐immolation across South Asia. Females predominated in almost all studies, with 100% of cases being female in Afghanistan [11] and in India [10], 80.64% in Pakistan [21], and 79.3% in Sri Lanka [23]. However, older studies from the 1990s had more male cases than female and both of the studies were conducted in India [16, 18]. The age distribution showed young adults as the most affected group, with mean ages ranging from 18.2 years in India [18] to 31 ± 16 years in India [17], whereas adolescents were a predominant age group in Afghanistan (55% aged 16–19 years) [11]. Motives that influenced to attempt self‐immolation were different among countries. Forced or child marriage (29%) and “badal” exchange marriage (bride‐for‐bride exchange) (18%) were the primary causes of self‐immolation in Afghanistan [11]. In contrast, one Indian study reported dowry‐driven violence against women as the motive in 100% of the cases examined [10]. Marital conflict and domestic dispute were the main motives for suicide in both Pakistan and Sri Lanka [21, 22, 23]. Psychosocial correlates, including low education, rural residence, family conflict, and oppression of women, were consistently reported in studies from India, Pakistan, Sri Lanka, and Afghanistan [11, 16, 18, 19, 20, 21, 22, 23], which highlighted the sociocultural vulnerability across South Asia.

### 3.8 Prevention and Policy Recommendations

Although most of the studies wrote about the harmful cultural practices of self‐immolation, few studies provided formal prevention strategies. Among those few, Raj et al. [11] and Kumar [10] suggested strengthening women's rights enforcement, education and empowerment programs, anti‐dowry legislation, and public awareness campaigns. A case‐based study recommended early screening for psychiatric disorders to women who suffered perinatal mental illness [20].

## 4 Discussion

### 4.1 The Major Findings of the Review

This review synthesized evidence on self‐immolation focusing on epidemiology, motives, and sociocultural contexts in South Asia. Previous studies focused on self‐immolation among females, as a protest form of suicides highlighting the sociocultural aspects of it [12, 13, 14]. This review describes the phenomenon holistically without restricting any specific gender and motive in the region. Self‐immolation was predominantly observed among females, particularly in late adolescence and early adulthood. The dominance of female self‐immolators is an indication of the abuse that women experience in these societies, with self‐immolation as the culturally scripted response to such abuse [12, 13, 14]. Similar findings were reported in Iran [24, 25]. Approximately 71% of self‐immolation cases in Iran involved young females [24]. As has been observed and summarized previously, these findings contrast sharply with the available data from HICs. In HICs (e.g., Italy, Germany, USA, and Canada), only 0.06% to 1% of all suicide deaths were caused by self‐immolation, and most of the self‐immolators were male [3, 4]. Moreover, most cases of self‐immolation in HICs involve Asian immigrants, and of these, 20% were Indian [3]. The mean age of self‐immolators ranged from 18.2 to 31 years, which may reflect the vulnerability of young adults who may struggle more than older cohorts to cope with increased marital, familial, and social injustice. With respect to age, similar findings were found in other studies globally, across countries. A meta‐analysis of 29 studies found the estimated average age was 27.3 years [26] and one study specifically noted that 60% of self‐immolation cases occurred in the 16–25 year age group [27].

### 4.2 Social Contexts

Marital conflict, domestic disputes, domestic violence, and dowry‐related issues were the most frequently reported social contexts of women's self‐immolation. Consistent with prior reviews [12, 13, 14], this review found that many cases involved young women experiencing systematic oppression and violations of their human rights, domestic violence, spousal abuse, forced or early marriage, and dowry‐related abuse who used self‐immolation as a form of protest against injustice. These circumstances highlight social inequalities, limited mental health support, and a lack of protection for women. In Afghanistan, forced or child marriages and “badal” marriages were the primary causative factors [11], whereas dowry‐related deaths accounted for all cases in one Indian study [10]. These findings were unique compared to other studies in the world. However, in the rest of the studies from India, Pakistan, and Sri Lanka, marital disputes, domestic conflict, unemployment, and poverty were the associated factors [16, 17, 19, 21, 22, 23] which is similar to other studies in a global context. One Iranian study found that marital conflict with a spouse increased self‐immolation risk by 7.8 times, and conflict with family members increased risk 10 times [27]. Male self‐immolators were more often associated with economic crisis, unemployment, political protests, or psychological disorders; however, their numbers were lower [28]. These findings emphasize that self‐immolation in South Asia is not merely an act of personal distress but frequently reflects structural gender inequality, systemic oppression of women, and broader human rights violations [12, 13, 14].

### 4.3 Methods and Outcomes

The consistent use of kerosene, open flames, and household fire sources across studies, as many have pointed out the immediate accessibility in the context of daily household life of these self‐immolation means in the countries under review here. Fatality rates were high, ranging from 70% to 100% in most studies, particularly among cases involving dowry‐driven violence and domestic violence [19, 21]. Conversely, self‐immolation related to protest or psychiatric illness demonstrated comparatively lower mortality [16], suggesting a different intention behind the act of self‐immolation. Self‐immolation in certain South Asian cases appears to be fundamentally a tool of protest or bargaining [12]. These patterns emphasized the importance of rapid intervention and accessibility to emergency care as potential modifiable factors to reduce mortality.

### 4.4 Psychological and Social Correlates

All the studies under review emphasize the influence of sociocultural factors primarily, violations of women's rights, including spousal abuse, domestic violence, coercive marital relations, economic dependency, denial of autonomy, low socioeconomic status, and dowry‐related pressures. Two previous studies also indicate that self‐immolation among females is associated with public adversities, including economic adversities—as a result of systemic discrimination against women in education, barriers for women to engage in paid work, discrimination against women in inheritance and ownership, and women's limited freedom of movement [6, 7, 12, 13, 14]. Similar findings were found in Iran and Algeria. In Algeria most of the self‐immolators engaged in self‐immolation as a protest against social injustices, such as discrimination and inequality, and these protests, in turn, were heavily influenced by the housing crisis and unemployment. Similarly, in Iran, Kurdish women usually chose self‐immolation due to social injustice, such as gender inequality, lack of support, and oppressive cultural expectations, along with poverty, generalized violence, divorce, relentless abuse, and coercive control [6]. Accounts from women who survived self‐immolation describe their actions as responses to chronic violence, ongoing abuse, loss of autonomy, and entrapment in human‐rights violations, rather than as acts driven solely by “humiliated ego” or wounded pride [12, 13, 14]. Psychiatric disorders, including personality disorders, dysthymia, and postpartum psychosis, were also reported as primary or contributing causes of suicide by self‐immolation. These findings are consistent with other studies and point to the inevitable interactive effect of social and individual mental health factors. An Iranian case–control study found that 67% of self‐immolation patients had psychiatric disorders [27]. The findings highlight the interaction between social, cultural, and psychological determinants, where structural inequalities increase individual distress, which, combined with the immediate household availability of means to perform the act, influences self‐immolation.

### 4.5 Cross‐Country Variations

Although female predominance and young adult vulnerability were common across South Asia, the underlying motives varied from one country to another. In Afghanistan, harmful cultural practices like child marriage and “badal” marriage were dominant causes, whereas dowry‐related violence was only reported in India. Marital conflict was a central factor in India, Pakistan, and Sri Lanka. This variation underscores the need for culturally sensitive interventions that consider local customs and specific societal pressures. Most of the studies were conducted in India, with limited studies from Pakistan, Sri Lanka, Afghanistan, and none from Bhutan, Bangladesh, or the Maldives. This suggested that due to underreporting and lack of research papers might hide the real number of cases and their causes in some countries.

### 4.6 Implications for Prevention and Policy

The findings of this review have clear implications for prevention. Strengthening enforcement of women's rights, anti‐dowry legislation, and public awareness campaigns have been suggested as critical structural interventions for some countries [10, 11, 12, 13, 14]. Human rights perspectives could be attempted to explore the association with self‐immolation. Early identification of psychiatric disorders, particularly among perinatal women, is also necessary to prevent future maternal suicide [20]. Gender‐sensitive community‐based interventions, combined with legal, social, and educational programs, could prevent the sociocultural vulnerabilities that led to female self‐immolation [12, 13, 14]. Additionally, ensuring access to prompt burn care services might reduce mortality among survivors.

### 4.7 Strengths and Limitations

This review provides a comprehensive synthesis of self‐immolation in South Asia that integrates demographic, psychosocial, and cultural perspectives. However, several limitations should be acknowledged. A limitation to drawing inferences is that most of these studies in this instance were conducted in India, with limited studies from Pakistan, Sri Lanka, Afghanistan, and none from Bhutan, Bangladesh, or the Maldives. Underreporting and a lack of research efforts in these countries undoubtedly have led to underreporting, obscuring the real number of cases and their causes in these countries and making comparisons limited. Moreover, most included studies were hospital‐based and cross‐sectional, which limited their generalizability to community settings. We were unable to perform a meta‐analysis due to heterogeneity in study design, sample size, and reporting methods. Very few studies have been conducted on this topic to identify the risk factors of self‐immolation in South Asia correctly. Our search terms may restrict some papers, as suicide by self‐immolation has been discussed in articles addressing suicide methods overall. Despite these limitations, the review highlighted the socio‐demography of the self‐immolators, risk factors, cross‐country variations, psychosocial correlations, and actionable insights for prevention.

## 5 Conclusion

Self‐immolation is a neglected public health and social issue in South Asia. This review found that most cases involved young women exposed to systematic oppression and violations of their human rights, domestic violence, spousal abuse, forced or early marriage, and dowry‐related abuse, who used self‐immolation as a form of protest against injustice. These cases highlight deep‐rooted social inequalities, limited mental health support, and a lack of protection for women. Further research is warranted to explore the psychological, cultural, human rights violations and other risk factors influencing self‐immolation so that effective prevention strategies could be developed. Preventive efforts should focus on raising public awareness, strengthening legal protections for women, reducing social stigma, improving mental health care services, ensuring human rights, and addressing sociocultural vulnerabilities that drive female self‐immolation.

## Author Contributions

Conception: S. M. Yasir Arafat and Sujita Kumar Kar. Data curation: Sadeed Hossain. Writing – original draft: S. M. Yasir Arafat and Sadeed Hossain. Writing – review and editing: S. M. Yasir Arafat, Sadeed Hossain, and Sujita Kumar Kar. All authors have read and approved the final version of the manuscript.

## Funding

The authors have nothing to report.

## Conflicts of Interest

The authors declare no conflicts of interest.

## Data Availability

The data that support the findings of this study are available from the corresponding author upon reasonable request.

## References

[1] R. Bidaki , S. Shirani , and M. Shamsian , “A Review of the Various Suicide Methods Used around the World,” International Journal of Medical Reviews 3, no. 4 (2016): 504–507, 10.15171/ijmr.2016.11.

[2] WHO . Suicide data (WHO, 2025). https://www.who.int/teams/mental‐health‐and‐substance‐use/data‐research/suicide‐data.

[3] A. Ahmadi , “Suicide by Self‐Immolation: Comprehensive Overview, Experiences and Suggestions,” Journal of Burn Care & Research 28, no. 1 (2007): 30–41, 10.1097/BCR.0b013E31802C8878. 17211198

[4] B. Poeschla , H. Combs , S. Livingstone , S. Romm , and M. B. Klein , “Self‐Immolation: Socioeconomic, Cultural and Psychiatric Patterns,” Burns 37, no. 6 (2011): 1049–1057, 10.1016/j.burns.2011.02.011. 21489697

[5] M. Cleary , J. Singh , S. West , M. Rahkar Farshi , V. Lopez , and R. Kornhaber , “Drivers and Consequences of Self‐Immolation in Parts of Iran, Iraq and Uzbekistan: A Systematic Review of Qualitative Evidence,” Burns 47, no. 1 (2021): 25–34, 10.1016/j.burns.2019.08.007. 31928787

[6] J. Y. Lebni , M. Mansourian , M. Hossain Taghdisi , B. Khosravi , A. Ziapour , and G. Demir Özdenk , “A Study of Kurdish Women's Tragic Self‐Immolation in Iran: A Qualitative Study,” Burns 45, no. 7 (2019): 1715–1722, 10.1016/j.burns.2019.05.012. 31202529

[7] S. M. Y. Arafat and S. Kar , “Epidemiology of Psychiatric Disorders and Overview of Access to Mental Health Care in South Asia,” in Access to Mental Health Care in South Asia (Springer, 2024), 10.1007/978-981-99-9153-2_1.

[8] World Bank , World Bank Country and Lending Groups—World Bank Data Help Desk (World Bank, 2023), https://datahelpdesk.worldbank.org/knowledgebase/articles/906519‐world‐bank‐country‐and‐lending‐groups.

[9] World Bank , World Bank Open Data (World Bank, 2023), https://data.worldbank.org.

[10] V. Kumar , “Burnt Wives–A Study of Suicides,” Burns: Journal of the International Society for Burn Injuries 29, no. 1 (2003): 31–35, 10.1016/s0305-4179(02)00235-8. 12543042

[11] A. Raj , C. Gomez , and J. G. Silverman , “Driven to a Fiery Death–the Tragedy of Self‐Immolation in Afghanistan,” New England Journal of Medicine 358, no. 21 (2008): 2201–2203, 10.1056/NEJMp0801340. 18499564

[12] S. S. Canetto , “Women's Protest Suicide by Self‐Burning in Southern and Central Asia: A Political Act to Decry Systemic Discrimination, Abuse and Violence,” BMC Women's Health 26, no. 1 (2025): 44, 10.1186/s12905-025-04028-z. 41350706 PMC12825254

[13] S. Canetto , S. Pouradeli , M. Khan , and M. Rezaeian , “Suicidal Self‐Burning in Women and Men Around the World: A Cultural and Gender Analysis of Patterns and Explanations,” in Suicide Risk Assessment and Prevention (Springer, 2022), 10.1007/978-3-030-41319-4_61-1.

[14] S. S. Canetto and M. Rezaeian , “Protest Suicide Among Muslim Women: A Human Rights Perspective,” in Suicide and Social Justice (Routledge, 2019).

[15] S. Ganesamoni , V. Kate , and J. Sadasivan , “Epidemiology of Hospitalized Burn Patients in a Tertiary Care Hospital in South India,” Burns: Journal of the International Society for Burn Injuries 36, no. 3 (2010): 422–429, 10.1016/j.burns.2009.06.212. 19782475

[16] V. P. Mahla , S. C. Bhargava , R. Dogra , and S. Shome , “The Psychology of Self‐Immolation in India,” Indian Journal of Psychiatry 34, no. 2 (1992): 108–113. 21776109 PMC2981043

[17] M. Natarajan , “Differences Between Intentional and Non‐intentional Burns in India: Implications for Prevention,” Burns: Journal of the International Society for Burn Injuries 40, no. 5 (2014): 1033–1039, 10.1016/j.burns.2013.12.002. 24433938

[18] S. P. Singh , P. J. Santosh , A. Avasthi , and P. Kulhara , “A Psychosocial Study of “Self‐Immolation” in India,” Acta Psychiatrica Scandinavica 97, no. 1 (1998): 71–75, 10.1111/j.1600-0447.1998.tb09966.x. 9504707

[19] S. A. Wagle , A. C. Wagle , and J. S. Apte , “Patients With Suicidal Burns and Accidental Burns: A Comparative Study of Socio‐Demographic Profile in India,” Burns: Journal of the International Society for Burn Injuries 25, no. 2 (1999): 158–161, 10.1016/s0305-4179(98)00138-7. 10208392

[20] M. U. Amjad and A. Zahra , “A Case of Self‐Immolation in a Woman with Recurrent Puerperal Psychosis from Pakistan,” BJPsych Open 10, no. S1 (2024): S271–S272, 10.1192/bjo.2024.651.

[21] M. Saaiq and B. Ashraf , “Epidemiology and Outcome of Self‐Inflicted Burns at Pakistan Institute of Medical Sciences, Islamabad,” World Journal of Plastic Surgery 3, no. 2 (2014): 107–114. 25489533 PMC4236991

[22] R. F. Fernando , M. Hewagama , W. D. Priyangika , S. Range , and S. Karunaratne , “A Study on Suicide by Self Immolation,” Ceylon Medical Journal 56, no. 4 (2011): 182–183, 10.4038/cmj.v56i4.3906. 22298219

[23] V. Laloë and M. Ganesan , “Self‐Immolation a Common Suicidal Behaviour in Eastern Sri Lanka,” Burns: Journal of the International Society for Burn Injuries 28, no. 5 (2002): 475–480, 10.1016/s0305-4179(02)00047-5. 12163288

[24] A. Ahmadi , R. Mohammadi , D. C. Schwebel , N. Yeganeh , A. Soroush , and S. Bazargan‐Hejazi , “Familial Risk Factors for Self‐Immolation: A Case‐Control Study,” Journal of Women's Health 18, no. 7 (2009): 1025–1031, 10.1089/jwh.2008.1192. 19558309

[25] A. Ahmadi , R. Mohammadi , D. Stavrinos , A. Almasi , and D. C. Schwebel , “Self‐Immolation in Iran,” Journal of Burn Care & Research: Official Publication of the American Burn Association 29, no. 3 (2008): 451–460, 10.1097/BCR.0b013e31817112f1. 18388564

[26] M. Parvareh , M. Hajizadeh , S. Rezaei , B. Nouri , G. Moradi , and N. Esmail Nasab , “Epidemiology and Socio‐Demographic Risk Factors of Self‐Immolation: A Systematic Review and Meta‐Analysis,” Burns 44, no. 4 (2018): 767–775, 10.1016/j.burns.2017.08.013. 29032973

[27] A. Ahmadi , R. Mohammadi , and A. Almasi , “A Case‐Control Study of Psychosocial Risk and Protective Factors of Self‐Immolation in Iran,” Burns 41, no. 2 (2015): 386–393, 10.1016/j.burns.2014.07.025. 25406886

[28] N. A. Pacha , “Algeria, From Social Issue to Self‐Immolation; Autopsy of a “Fashionable” Suicide,” European Psychiatry 41, no. S1 (2017): S884–S884, 10.1016/j.eurpsy.2017.01.1788.

